# Surgical packages for laparoscopic surgery

**DOI:** 10.4103/0972-9941.16531

**Published:** 2005-06

**Authors:** K. Bhattacharya

**Affiliations:** Department of Surgery, Sri Ramachandra Medical College and Research Institute, Deemed University, Chennai, India

**Keywords:** Laparoscopic surgery, learning curve, package system

## Abstract

‘Packages’ are in fashion today for most surgical procedures in various corporate hospitals and this has included laparoscopic procedures too. A package system enables the hospitals to get cost settlements done more easily. Also, it is more convenient for the patients who are aware upfront of the charges. The principal disadvantages seems to be for the surgeon, who may face displeasure of the patient, hospital or insurance agencies apart from forfeiting his personal charges if (a) he is a novice in laparoscopic surgery and takes extra time to complete a procedure, (b) unforeseen problems occur during surgery, or (c) new pathologies are discovered on exploration.

## INTRODUCTION

A majority of the private and corporate hospitals have designed various packages for the laparoscopic surgeries performed in the hospital. According to the central government health scheme (CGHS), a package is defined as a lump sum cost of inpatient treatment for which a patient has been referred by a competent authority or CGHS to the hospital or diagnostic center. This includes all the charges pertaining to a particular treatment/procedure including registration charges, admission charges, accommodation charges, injection/transfusion charges, dressing charges, operation charges, anesthetic charges, operation theatre charges, procedural charges/surgeon fee, doctor/consultant visit charges, ICU/ICCU charges, monitoring charges, cost of disposable surgical charges and cost of all sundries used during hospitalization-related routine investigation, and physiotherapy charges, etc. from the time of admission to the time of discharge.[1] The only charges that are excluded from the package are telephone charges and toiletries charges. The insurance companies and government organizations like the CGHS referring these patients for laparoscopic surgery to such private/corporate hospitals insist on strict adherence to the packages and routinely block any charges if they exceed the schedule package, irrespective of any valid reason offered by the hospital or due to any unforeseen complication. A time has come to ponder over this issue and debate whether such packages are really doing ‘good’ to the patient. It is certainly not giving a ‘feel good’ factor to the minimally invasive surgeons.

## ADVANTAGES OF THE PACKAGE SYSTEM

The package system was forced upon the hospitals by various government establishments and insurance companies when it was realized that there is a great variation in the cost of laparoscopic surgery in different hospitals in various corners of the country. A laparoscopic cholecystectomy at smaller nursing homes may cost around Rs. 10 000, which may be double if performed at large private hospitals.[2] The package system ensures uniformity of surgical expenses, less inconvenience for the patient and the company's approval for the surgery. It is time to question as to how the packages are fixed. A few bureaucrats and doctors, based on the basic salary of the patient or his dependent, decide as to what package he is entitled to in the general/special ward. In most of the cases, the packages are faxed to the concerned employee and the money for the surgical expenses is sanctioned even before the surgeon has wheeled in the patient to the operation theatre.

## DISADVANTAGES OF THE PACKAGE SYSTEM

Whenever a patient is referred under the ‘package’ system, the surgeon is in a difficult position. For example, if the duration of surgery increases due to unexpected operative findings such as acute cholecystitis with adhesions in the Calot's triangle. A complex situation arises when the patient who is referred undergoes multiple surgeries. For example, when the authority refers a patient for laparoscopic cholecystectomy, his gallbladder alone must be removed and in no way is the surgeon allowed to discover/tamper with his inflamed appendix. In essence, this is seen as a deterrent to a thorough diagnostic laparoscopy as it may bring forth a ‘nonpackage’ pathology, which may require intervention. Even if the surgeon decides to treat the other pathology discovered, his own surgical fees are likely to be deducted by the hospital if the referring authority/insurance company refuses to sanction any excess charges for the extra procedure. Further, it is expected that surgeons should have no learning curve, have a nil complication rate and there should be no patient-related morbidity during laparoscopy. The duration or dosage of antibiotics may not be increased as it may increase the charges beyond the sanctioned package. The patient is expected to be discharged within 72 hours irrespective of his medical condition. During surgery, one has to be doubly careful during clipping the cystic artery and cystic duct as the ‘quota’ is one clip pack only during laparoscopic cholecystectomy. If laparoscopic surgery is converted to an open procedure, the company sanctions for one package only. Thus, the surgeon may be forced into proceeding with laparoscopy in an effort to avoid a conversion – a dangerous situation indeed!

## CONCLUSIONS

Although laparoscopic surgery carries well-defined benefits and usually does not cross the package charges, the competent authority should be liberal in cases where there have been unexpected developments, especially in minimally invasive surgery. As many of the younger surgeons are still in their learning curve and the majority of the medical colleges are yet to teach laparoscopic surgery in their surgical curriculum, it is but natural that complications will occur irrespective of the experience and qualification of the surgeon. The package system should be such that untoward/unforeseen complications or prolonged antibiotics or hospitalization, if needed, are taken care of without penalizing the patient, surgeon, or the hospital. In a landmark study on the cost analysis of laparoscopic cholecystectomy, it was concluded that the areas in which hospitals and surgeons can improve the surgical value package (i.e., decrease costs while maintaining quality) are disposable equipment, and efficient reduction in the theatre time.[3] Hospitals can maximize their returns on minimally invasive surgery by providing ample training to the surgical and processing staff, by standardizing equipment, by wisely choosing when to use custom kits, block scheduling for minimally inversive surgery (MIS) and incorporating MIS into the hospital's strategic plan.[4] According to Traverso, the concept of increasing value by increasing quality without an attempt to decrease costs is a very important principle that the health care system must learn in our everchallenging medical environment.[5] Until then, it is recommended that the surgeon should be selective in choosing his patient for laparoscopic surgery. Only those who are fit and in ASA Grade 1 should be included in the package system to derive the benefits of the system.

‘There are coaches who spend 18 hours a day coaching the perfect game. They still lose some games because the ball is oval and they cannot control the bounce.’

– Bud Grant (Ex-coach of Minnesota Vikings)
